# Women's COPD

**DOI:** 10.3389/fmed.2021.600107

**Published:** 2022-01-03

**Authors:** Maéva Zysman, Chantal Raherison-Semjen

**Affiliations:** ^1^Centre de Recherche cardio-thoracique de Bordeaux, Université de Bordeaux, Pessac, France; ^2^Service des Maladies Respiratoires, CHU Bordeaux, Pessac, France; ^3^Epicene U1219, Université de Bordeaux, Bordeaux, France

**Keywords:** chronic obstructive pulmonary disease (COPD), gender, epidemiology, smoking, perspective

## Abstract

Chronic obstructive pulmonary disease (COPD) is no longer a respiratory disease that predominantly affects men, to the point where the prevalence among women has equaled that of men since 2008, partly due to their increasing exposure to tobacco and to biomass fuels. Indeed, COPD has become the leading cause of death in women in the USA. A higher susceptibility of female to smoking and pollutants could explain this phenomenon. Besides, the clinical presentation appears different among women with more frequent breathlessness, anxiety or depression, lung cancer (especially adenocarcinoma), undernutrition and osteoporosis. Quality of life is also more significantly impaired in women. The theories advanced to explain these differences involve the role of estrogens, smaller bronchi, impaired gas exchange in the lungs and smoking habits. Usual medications (bronchodilators, ICS) demonstrated similar trends for exacerbation prevention and lung function improvement in men and women. There is an urgent need to recognize the increasing burden of COPD in women and therefore to facilitate global improvements in disease management (smoking cessation, pulmonary rehabilitation…) in half of the population. Nevertheless, important limitations to the treatment of women with COPD include greater under-diagnosis than in men, fewer spirometry tests and medical consultations. In conclusion there is an urgent need to recognize the increasing burden of COPD in women and therefore to facilitate globally improvements in disease management in this specific population.

## COPD Epidemiology

Chronic obstructive pulmonary disease (COPD) is no longer a disease that exclusively affects men as its prevalence represents 9.23% [8.16–10.36] in men and 6.16% [5.41–6.95] in women of the whole population (1). In some countries, the prevalence of COPD is even higher in women than in men (2), and its prevalence increases more rapidly in women, particularly in younger women (3). Besides, women are more likely to be diagnosed and considered as asthmatic (4).

Whether gender confers a particular susceptibility to develop COPD has been controversial (5, 6), but there is increasing evidence suggesting that for a given level of risk exposure, women are more susceptible to developing COPD or to have faster disease progression than men (7, 8). For example, female smokers are at greater risk of airflow obstruction than male smokers (9, 10).

The increase in prevalence of COPD among women is also due increased exposure to tobacco and to biomass fuels (11, 12). In addition, specific professional exposure is not well known (13). For example, regular use of chemical disinfectants may lead to COPD development (14) and women with lower cumulative exposure years to aromatic solvents than men have a greater lung function decline than men (15).

## Clinical Presentation

### In Stable Condition

The clinical manifestations of COPD can differ by gender, with women experiencing worse lung function and health-related quality of life than men.

There is evidence of a higher susceptibility of female to smoking. Among more than 13,000 subjects, Prescott *et al*. estimated that the excess loss of FEV1 per pack-year of smoking was between 7.4 and 10.5 ml in female smokers compared to 6.0 to 8.4 ml in male smokers (16). The annual decline in lung density is quicker in women (17). Besides, there is a greater prevalence of females among those with early onset of COPD (6).

Women tend to report more symptoms given the same disease severity. For a given airflow obstruction and age, women with COPD more often exhibit anxiety (anxiety score being higher 9.8 vs. 7.1) (18–20). Quality of life is also more impaired in women (SGRQ scores 50.6 vs. 45.4; *p* < 0.02) (19). A higher burden of the disease with a higher dyspnea has been confirmed even after matching on age and FEV_1_ (18, 21, 22). However women are less likely to report cough and sputum (22) but for a given level of airflow obstruction, experience a greater impact on exercise capacity, breathlessness and poorer quality of life (Table 1).

**Table 1:** Clinical presentation differentiating COPD expression among men and women.

	**Women**	**Men**
Lung function decline	++	+
Exacerbations	++	+
Breathlessness	++	+
Cough/sputum	+	++
Quality of life	↓↓	↓
Comorbidities	Anxiety, depression, osteoporosis	Cardiovascular diseases
Underdiagnosis of COPD	+++	++

*COPD, Chronic Obstructive Pulmonary Disease. +: a little increased; ++: increased; +++: very increased; ↓: decreased*.

Comorbidities are also different between men and women in several studies (23, 24) with a higher frequency of anxiety, depression, osteoporosis in women but a lower prevalence of cardiovascular diseases compared to men (25).

### Exacerbations

Higher risk of exacerbation is reported among women as compared to men (18, 21, 22). Additionally, the risk of a first moderate or severe exacerbation was estimated to be 17% greater in women than in men (hazard ratio, 1.17; 95% CI, 1.12–1.23), with a median time to first exacerbation of 504 days for women and 637 days for men. As previously mentioned, COPD starts earlier in women explaining why these differences were more prominent in the younger age group from 40 years to 65 years (Table 1) (26). Moreover, longitudinal data in COPDGene revealed that female sex is independently associated with risk of acute episodes of respiratory disease independent of other relevant covariates (27). Therefore, women, compared with men, seem to be at greater risk for development of COPD and may present with more severe disease. Besides, females have an increased risk of hospitalization for COPD compared with males (RR = 1.5 [1.2; 2.1] to 3.6 [1.4; 9] (16, 28). Further, female representation is increasing among hospitalized COPD exacerbation over time (29). However male gender as compared to female seems to be associated with a poorer prognosis with a higher 30 and 90 day mortality (30).

## Treatments

Several limitations exist concerning treatments' response according to gender. First, a bias in physicians' awareness of COPD, which results in a higher rate of mis- or delayed-diagnosis in women with COPD compared with men, potentially leading to suboptimal treatment (31, 32). Although the gender bias in diagnosis is reduced by the use of spirometry, this tool remains underused, particularly in women (33, 34). Second, gendered analyses are dramatically lacking in pharmacologic trials and studies are underpowered for analyses based on sex, particularly when women are underrepresented.

Even if women with COPD are more likely to have interactions with healthcare providers than men (32, 35), they receive fewer classes of medications and non-pharmacologic interventions for COPD than men (22, 33, 36). Paradoxically, male are more likely to receive more than one maintenance drug, especially long-acting anti-muscarinic or dual long acting bronchodilators for COPD (37, 38).

While, smoking cessation is the most important initial step in COPD management, males seem to have higher sustained quit rates at 12 and 36 months. After adjusting on confounding biases, such as educational level, the effect of gender was lost at 12 months but remained significant at 36 months. Besides, specific drugs to help smoking cessation appear to be prescribed more often in women (37). and smoking cessation strategies appear to be more frequently proposed to women (39). This could imply that women have greater difficulty in sustaining long-term abstinence from tobacco than men (40). However there are few studies specifically designed to examine sex-related differences. Some nicotine-replacement therapies may not be as effective in women, in reducing craving less effectively in women than in men, resulting in larger weight gain in females. However, a study showed that bupropion was equally effective in females and males with COPD (41).

Pulmonary rehabilitation is also a key non-pharmacologic intervention for patients with COPD, but most studies enrolled more males than females and no differences have been shown between men and women. However, women seem to report more benefits than men in the dyspnea domain (+0.85 vs. 0.4 unit) and fatigue domain (0.55 vs. 0.3) (42). These benefits and the specific interventions that can maximize benefit for men and women require further research.

Finally in pharmacological trials, women are underrepresented and little is known about gender differences with respect to response to treatment. *Post-hoc* analyses of clinical trials assessing long-acting bronchodilators have shown similar trends for exacerbation prevention and lung function improvement in men and women (43, 44).

## Discussion

Many areas of the effects of gender on COPD pathogenesis, progression, presentation, and responses to treatments are still very poorly understood. Significant differences between countries and exposures influence airway disease development, phenotypes and diagnosis, especially COPD. Interestingly, among a huge cohort of 68,532 individuals within the Medicare Advantage plan, 22.8% had a diagnosis of COPD associated to asthma. Compared with COPD patients without asthma, there was a higher proportion was female (45).

These factors require much more detailed analyses and carefully performed prospective studies are needed to fully assess the effects of treatment in real world settings. In fact, several limitations exist concerning treatments' response according to gender and globally, gendered analyses are dramatically lacking in pharmacologic trials because studies are underpowered for analyses based on sex, particularly when women are underrepresented. The reasons for these observations are incompletely understood but highlight the importance of evaluating sex differences in COPD. There is a great challenge to better understand the impact of gender in the outcomes of COPD.

## Conclusion

Chronic obstructive pulmonary disease (COPD) is no longer a respiratory disease that predominantly affects men. Females seem to be more susceptible to the deleterious effects of smoking and have more impaired quality of life, more frequent exacerbations but less cardiovascular comorbidities. There is a need for further work in this area to determine how women with COPD can best be supported. Further research could focus on personalized approaches to achieve optimal treatment in women with COPD.

## Author Contributions

MZ and CR-S made substantial contributions to conception and design, acquisition of data, analysis and interpretation of data, involved in drafting the manuscript or revising it critically for important intellectual content, and gave final approval of the version to be published. Both authors contributed to the article and approved the submitted version.

## Conflict of Interest

The authors declare that the research was conducted in the absence of any commercial or financial relationships that could be construed as a potential conflict of interest.

## Publisher's Note

All claims expressed in this article are solely those of the authors and do not necessarily represent those of their affiliated organizations, or those of the publisher, the editors and the reviewers. Any product that may be evaluated in this article, or claim that may be made by its manufacturer, is not guaranteed or endorsed by the publisher.
